# Effect of MyTeen SMS-Based Mobile Intervention for Parents of Adolescents: A Randomized Clinical Trial

**DOI:** 10.1001/jamanetworkopen.2019.11120

**Published:** 2019-09-11

**Authors:** Joanna Ting Wai Chu, Angela Wadham, Yannan Jiang, Robyn Whittaker, Karolina Stasiak, Matthew Shepherd, Chris Bullen

**Affiliations:** 1 The National Institute for Health Innovation, School of Population Health, University of Auckland, Auckland, New Zealand; 2 Department of Psychological Medicine, Faculty of Medical and Health Sciences, University of Auckland, Auckland, New Zealand; 3 School of Psychology, Massey University, Auckland, New Zealand

## Abstract

**Importance:**

There is global pressure to respond to the burden posed by adolescent mental health problems. The National Mental Health Commission has made a call for investment in mobile health services directed at prevention and early intervention to relieve the demand on targeted mental health services that are costly to provide. Parents and primary caregivers play a significant role in the lives of adolescents and are important targets for such efforts. Currently, there is no evidence for the effectiveness of programs delivered solely via text message for parents of adolescents.

**Objective:**

To evaluate the effects of a text-messaging program (MyTeen) on promoting parental competence and mental health literacy for parents of adolescents.

**Design, Setting, and Participants:**

A parallel 2-group randomized clinical trial was conducted in New Zealand. A total of 221 parents and primary caregivers of adolescents aged 10 to 15 years were recruited from March 19 to August 17, 2018, via community outreach and social media and were randomly allocated 1:1 into the control or the intervention group. Statistical analysis was performed on the principle of intention to treat with adjustment for baseline factors and ethnicity.

**Intervention:**

A text-messaging program for parents of adolescents (age 10-15 years) to promote parental competence and mental health literacy. Participants received 1 daily text message over 4 weeks.

**Main Outcomes and Measures:**

Parental competence, assessed at 1 month after randomization by the Parenting Sense of Competence Scale.

**Results:**

In total, 221 participants (214 [96.8%] female) were randomized, 109 to the intervention group and 112 to the control group; 201 participants (91%) completed the trial at 3 months. Significant group difference was observed on the primary outcome at the end of 1 month of intervention, with participants reporting a higher level of parental competence than those in the control group (estimated mean difference, 3.33 points; 95% CI, 1.37-5.29 points; *P* = .002). Except for knowledge about mental health, all secondary outcomes were significant, including continued improvement in parental competence at 3 months (estimated mean difference, 4.08 points; 95% CI, 1.96-6.20 points; *P* < .001), knowledge of help seeking (estimated mean difference, 0.99 points; 95% CI, 0.49-1.50 points; *P* < .001), parental distress (estimated mean difference, −2.39 points; 95% CI, −4.37 to −0.40 points; *P* = .02), and parent-adolescent communication (estimated mean difference, 2.21 points; 95% CI, 0.48-3.95 points; *P* = .01), with participants in the intervention group reporting better parenting-related outcomes than the control group at 1 and 3 months after the intervention.

**Conclusions and Relevance:**

This text-messaging program for parents of adolescents appears to be an effective and feasible way to facilitate the implementation and delivery of evidence-based information to populations that are not easily reached with other intervention modalities. The program can be easily scaled up for delivery as an early preventive intervention and may represent a less expensive option for service delivery.

**Trial Registration:**

anzctr.org.au Identifier: ACTRN12618000117213

## Key Points

**Question:**

Is a brief parenting program delivered solely via text message effective in improving parental competence and mental health literacy in parents of adolescents?

**Findings:**

In this randomized clinical trial of 221 parents and caregivers, significant group differences were observed between those receiving the text-messaging intervention and the control group. Participants who received the text-messaging program reported higher levels of parental competence, improved knowledge of help seeking, improved parent-adolescent communication, and lower levels of parental distress at 1 and 3 months of follow-up compared with the control group.

**Meaning:**

The text-messaging program appears to be an effective and feasible way to reach and support a large number of parents to improve parental competence and may represent a less expensive option for service delivery.

## Teaser

This randomized clinical trial evaluates the effectiveness of a text-messaging program, MyTeen, for promoting parental competence and mental health literacy for parents of adolescents.

## Introduction

Adolescent mental health is a growing concern globally.^1,2,3^ In New Zealand, depressive disorder is a major health issue among adolescents, with prevalence rates of 4% to 8% at age 15 years increasing to 17% to 18% by age 18 years.^3^ The serious developmental consequences of adolescent depression, and associated treatment challenges once problems have developed, underscore the need for programs aimed at prevention.^4,5^

International policies recognize the importance of parents and caregivers in the prevention of depression and promotion of youth mental well-being.^6,7^ A recent meta-analysis^8^ reported that preventive interventions primarily targeting parents were associated with sustained improvements in children’s well-being that lasted up to 11 years after the intervention. However, compared with interventions directed at parents of young children, few interventions are available for parents of adolescents; more research is needed in this area.^9^ Of those available, most interventions have been found to be associated with improved parent-adolescent relationships, parenting practices, parent and adolescent well-being, and family functioning.^4,8,10,11^ Efforts that effectively improve parenting practices have great potential in improving youth outcomes.

However, uptake of parenting programs is poor, limiting their public health impact.^4,12,13^ Parent attendance rates for those who enroll in family-based programs typically range from 35% to 50% of sessions and up to one-third of those who sign up attend no sessions. This may be the result of many programs, which typically involve face-to-face participation, not aligning with the demands of family life.^13,14,15,16^ Hence, there is a need to engage families in a way that is not only convenient and accessible, but also addresses some of the barriers that preclude involvement at the outset (eg, stigma, cost, lack of awareness, and distrust of the system or professionals).

Mobile health (mHealth) interventions, particularly text-messaging programs, offer promise in this area^12^ owing to their simplicity and minimal participant burden. Text messaging requires basic, low-cost mobile phones, which reduces potential socioeconomic inequality of access. While there is evidence to suggest the feasibility and effectiveness of text-messaging interventions to address health issues,^17,18,19^ their application and impact on parents is unknown. There is considerable potential in New Zealand to supplement and enhance existing services for families by leveraging mHealth technologies in delivering programs for parents. Ninety-two percent of New Zealand households have access to a mobile phone,^20^ with no differences in internet access or smartphone ownership by ethnicity or education, and few differences by age younger than 65 years.^20^ The aim of this study was to evaluate the effectiveness of a text messaging-based mobile intervention (MyTeen) for parents of adolescents. We hypothesized that participants who received the program would report higher levels of parental competence and mental health literacy compared with the control group.

## Methods

### Study Design

We conducted a parallel 2-group randomized clinical trial between March 19 and August 17, 2018, in which eligible parents and primary caregivers (hereafter referred to as *parents*) were randomly allocated to the MyTeen program or care as usual (control group). Data were obtained from all participants at baseline, 1 month (end of intervention phase), and 3 months after randomization. The study protocol is available in Supplement 1 and has been published elsewhere.^21^ The study was approved by the University of Auckland Human Participants Ethics Committee. All participants were given information about the study and gave verbal and electronic consent to be included. This study followed the Consolidated Standards of Reporting Trials (CONSORT) reporting guideline.

Parents were recruited nationwide in New Zealand via social media, distribution to organizational (eg, family services, community organizations) emailing lists, schools, and word of mouth. Parents were eligible for inclusion if they indicated at screening that they had an adolescent child aged 10 to 15 years, owned a mobile phone, were not currently receiving professional assistance for their own and/or their child’s mental health problems, could read and understand English, and were willing to participate and provide information at scheduled follow-up points. Only 1 parent from each household was invited to participate. Parents who did not meet the inclusion criteria or reported a high level of stress based on the Parental Stress Index (ie, ≥72 points) at screening were excluded; the latter were directed to professional services. Power analysis indicated that to detect a mean (SD) group difference of 2.5 (5.8) in the Parenting Sense of Competence (PSOC) scale score at 1-month follow-up and allowing for an estimated 20% loss to follow-up with 80% power, 214 participants (107 per randomized group; 1 parent per household) were needed.

### Study Procedures

Potential participants expressing interest were contacted by our research assistant, who explained the study and screened for eligibility. Eligible participants were sent an email with the participant information sheet, consent form, and link to the first online questionnaire. Randomization took place once the baseline assessment was completed online, with eligible participants randomly allocated 1:1 to either the intervention or the control group. Participants were notified via email which group they were allocated to. Those randomized into the intervention received the program the day after the notification email. Control participants received no intervention from the research team but could access alternative services if they wished. The randomization sequence was computer generated by the trial statistician (Y.J.) using block randomization with variable block sizes of 2 or 4 and stratified by Māori, Pacific, and non-Māori/non-Pacific ethnic groups. The randomization lists were concealed in a secure database until the point of randomization. Owing to the nature of the intervention, it was not possible to blind participants or research staff to the allocated treatments.

At 1 and 3 months after randomization, participants in both groups received emails directing them to complete the 1- and 3-month follow-up assessments. Reminder emails and/or text messages were sent when participants did not complete the assessments. A follow-up telephone call was made to the participant if the assessment was not completed after 2 reminders. Upon completion of the 3-month follow-up, the program was offered to control participants. Each participant received a $NZD20 gasoline voucher to recognize their participation and entered into a drawing for a $NZD150 supermarket voucher.

### Study Intervention

MyTeen is a tailored program of text messages (≤160 characters in length) with evidence-based parenting tips for establishing and maintaining positive relationships with adolescents; strategies to increase adolescent autonomy; advice about adolescent development, family functioning, and parental self-care; information to help parents recognize depressive symptoms and understand treatment options; and links to evidence-based support and informational resources (eTable in Supplement 2). The text messages were adapted from the Parenting Strategies Program,^22^ a set of evidence-based parenting guidelines developed through (1) systematic review and meta-analysis of parental factors associated with adolescent depression and/or anxiety and (2) a Delphi study of international expert consensus about actionable strategies parents can use to reduce their child’s risk of depression and anxiety. The guideline has been evaluated and found to be useful as a universal prevention strategy for parents of adolescents.^23^ We undertook formative work with focus groups of parents to ensure that the content, intensity, and duration of the intervention were appropriate, acceptable, and feasible. As a result, we scheduled once-daily free text messages to participants at a time of day they selected at the screening call for a 4-week period. Participants were able to discontinue the program at any time by replying “stop” to the messages received.

### Measures

At baseline, we collected demographic data including parents’ sex, marital status, ethnicity, education level, and employment status. Data on household income, family structure, and child’s age, sex, and ethnicity were also recorded.

The primary outcome was parental competence, assessed at 1 month after randomization by the PSOC Scale,^24^ which measures parental self-esteem on 2 dimensions: satisfaction (feelings associated with parenting, as anxiety or frustration) and efficacy (perceived ability and confidence in handling parenting problems). These constructs are closely linked with both positive family interactions and positive child development. The total PSOC score is calculated as the sum of 17 items and has a possible range of 17 to 102. The PSOC has substantial strengths, including good content validity, internal consistency (α = .80), normative data, test-retest reliability (0.73-0.74),^25^ and indicators of both convergent and discriminant validity.

Secondary outcomes included continued improvement of PSOC score at 3-month follow-up, knowledge of mental health issues, parental distress, and parent-adolescent communication. Knowledge of mental health issues was measured by a 7-item scale, developed by Fox,^26^ at 1- and 3-month follow-up, in which parents rate agreement or disagreement with the items to create a score from 0 to 7, where a higher number indicates greater knowledge of depression. Knowledge of where to seek information from the Mental Health Literacy Scale^27^ was also evaluated at 1- and 3-month follow-up. The subscale consists of 4 items, rated on a 5-point scale, ranging from strongly disagree to strongly agree*.* The scale has demonstrated good internal and test-retest reliability, and scores are significantly correlated with help-seeking intentions. Parental distress was measured at 1- and 3-month follow-up by the Parental Stress Scale,^28^ which consists of 18 items rated on a 5-point scale from strongly disagree to strongly agree to generate a total score. The scale has good internal reliability (0.83) and test-retest reliability (0.81). Parent-adolescent communication was measured at 1- and 3-month follow-up by the Parent Adolescent Communication Scale.^29^ This scale consists of 20 items and generates a total score and 2 subscale scores (open family communication and problems in family communication). The scale has good internal reliabilities for both subscales (0.87 and 0.78, respectively) and test-retest reliabilities (0.78 and 0.77, respectively). Program satisfaction was measured at 1 month and completed by the intervention group only.

### Statistical Analysis

Statistical analyses were performed using SAS statistical software version 9.4 (SAS Institute), with all tests 2-sided at the 5% significance level. Baseline data from all randomized participants were summarized by treatment group. Continuous variables are presented as means and standard deviations and categorical variables as frequencies and percentages. Primary and secondary outcomes were summarized descriptively by treatment group at each point. The primary outcome analysis used the intention-to-treat (ITT) population that included all randomized participants. The main effect of intervention was tested using linear regression adjusting for baseline outcome value and ethnicity (stratification factor) in the model. Adjusted group difference and 95% confidence intervals were reported with associated *P* value. The consistency of treatment effects between ethnic groups was tested using an interaction term with treatment group in the main model. Missing data were imputed using multiple imputations in the primary ITT analysis, which created multiple imputed data sets (n = 50) that were analyzed using the same regression models and combined for 1 inference. The Markov Chain Monte Carlo method was used to estimate the group difference assuming the data were from a multivariate normal distribution and missing at random. We did sensitivity analysis to explore the impact of missing data using the observed data only and a per-protocol analysis that excluded participants with major protocol violations.

Similar regression analyses were carried out on other secondary outcomes measured at 1- and 3-month follow-up without imputation. As a secondary approach, linear mixed models were used on the outcomes measured at both points, controlling for repeated measures on the same participant using a random participant effect. Missing data were considered in modeling assuming they were missing at random. The robustness of treatment effects using different regression models was assessed.

## Results

Of 319 individuals expressing interest in the study, 236 were screened for eligibility and 221 were randomized to the intervention (n = 109) or control (n = 112) groups (Figure). Attrition was low at 1 month (4.5% overall; 5 participants [4.6%] in the intervention group and 5 [4.5%] in the control group) and at 3-month follow-up (9% overall; 10 participants [9.2%] in the intervention group and 10 [8.9%] in the control group). Overall, 201 participants (91%) had completed the trial at 3 months. One major protocol violation was reported for a participant randomized into the wrong strata due to an error in coding the participant’s ethnicity.

**Figure.**
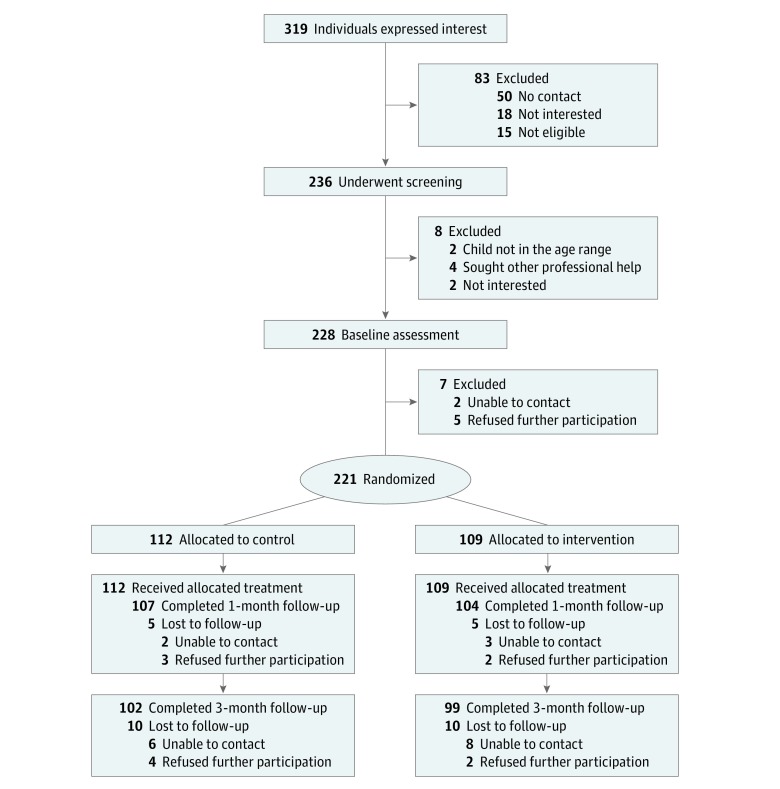
CONSORT Diagram Detailing Participant Flow

### Participant Characteristics

The study cohort consisted of 221 parents of adolescents (214 [96.8%] female), of whom 210 (95.0%) were mothers (including stepmother), 7 (3.2%) were fathers, 3 (1.4%) were grandparents, and 1 (0.4%) was a close relative. The majority (75.6%) of participants identified themselves as European followed by Māori (Indigenous New Zealanders; 13.1%), Pacific (7.7%), and other ethnicity (3.6%). More than half of the adolescents were male (54.8%), with a mean (SD) age of 12.30 (1.55) years in the control group and 12.28 (1.65) years in the intervention group. A majority (82.8%) of participants were either married or living with their partner and 168 (76.0%) had a college degree (Table 1).

**Table 1.** Baseline Characteristics of Participants

Characteristic	No. (%)	
	Control (n = 112)	Intervention (n = 109)
Sex		
Male	3 (2.7)	4 (3.7)
Female	109 (97.3)	105 (96.3)
Ethnicity		
New Zealand European	71 (63.4)	69 (63.3)
Māori	15 (13.4)	14 (12.8)
Pacific	9 (8.0)	9 (8.3)
Asian	4 (3.6)	4 (3.7)
Other	13 (11.6)	13 (11.9)
Ethnicity stratification		
Māori	15 (13.4)	14 (12.8)
Pacific	9 (8.0)	8 (7.3)
Non-Māori/non-Pacific	88 (78.6)	87 (79.8)
Marital status		
Married or cohabiting	91 (81.3)	92 (84.4)
Divorced, separated, or widowed	16 (14.3)	14 (12.8)
Never married	5 (4.5)	3 (2.8)
Household income, NZD^a^		
<15 000	1 (0.9)	0
15000-29 999	2 (1.8)	0
30 000-59 999	12 (10.7)	12 (11.0)
60 000-99 999	30 (26.8)	35 (32.1)
≥100 000	55 (49.1)	57 (52.3)
Decline to say or don’t know	12 (10.7)	5 (4.6)
Child’s age, y		
10	17 (15.2)	21 (19.3)
11	21 (18.8)	19 (18.4)
12	24 (21.4)	19 (17.4)
13	21 (18.8)	20 (18.3)
14	19 (17.0)	18 (16.5)
15	10 (8.9)	12 (11.0)
Child’s sex		
Male	64 (57.1)	57 (52.3)
Female	48 (42.9)	52 (47.7)
Relationship to the child		
Mother	105 (93.8)	103 (94.5)
Father	3 (2.7)	4 (3.7)
Stepparent	1 (0.9)	1 (0.9)
Grandparent	3 (2.7)	0
Close relative	0	1 (0.9)
Household structure		
Original family	79 (70.5)	80 (73.4)
Stepfamily	10 (8.9)	10 (9.2)
Sole parent family	15 (13.4)	15 (13.8)
Living with extended family	6 (5.4)	3 (2.8)
Other	2 (1.8)	1 (0.9)

Abbreviation: NZD, New Zealand dollars.

^a^ One NZD is equivalent to 0.64 US dollar.

### Primary Outcome

The primary ITT analysis showed a significant estimated mean difference of 3.33 points (95% CI, 1.37-5.29 points; *P* = .002) between the groups at the end of the 1-month intervention, with the intervention group reporting a significantly higher level of parental competence than the control group (Table 2). Sensitivity analysis based on complete cases (estimated mean difference, 3.36 points; 95% CI, 1.50-5.23 points; *P* < .001) and per-protocol analysis (estimated mean difference, 3.32 points; 95% CI, 1.45-5.19 points; *P* < .001) showed similar results to the main analysis.

**Table 2.** Raw Scores and Group Differences for Primary Outcome at 1 Month

Measure	ITT With Multiple Imputations (n = 221)								Estimated Mean Difference (95% CI)			
	Raw Score, Mean (SD)				Estimated Mean Difference (95% CI)	*P* Value	Standardized Mean Difference (95% CI)	*P* Value				
	Control (n = 112)		Intervention (n = 109)						Per Protocol (n = 210)	*P* Value	Complete Cases (n = 211)	*P* Value
	Baseline	1 mo	Baseline	1 mo								
Parenting Sense of Competence Scale	71.8 (10.1)	70.8 (10.9)	69.6 (9.07)	72.4 (8.6)	3.33 (1.37-5.29)	.002	0.34 (0.14-0.54)	<.002	3.32 (1.45-5.19)	<.001	3.36 (1.50-5.23)	<.001

Abbreviation: ITT, intention to treat.

### Secondary Outcomes

Results on all secondary outcomes at each assessment are presented in Table 3. Significant group differences were observed at 1 month with parents in the intervention group reporting improved knowledge of help seeking (estimated mean difference, 0.95 points; 95% CI, 0.44-1.45 points; *P* < .001) and improved parent-adolescent communication (estimated mean difference, 2.13 points; 95% CI, 0.64-3.62 points; *P* = .005). Parents in the intervention group reported significantly more open communication with their adolescent than parents in the control group (estimated mean difference, 1.47 points; 95% CI, 0.61-2.33 points; *P* < .001). No significant differences were observed for knowledge of mental health issues (estimated mean difference, 0.19 points; 95% CI, −0.11 to 0.48 points; *P* = .21) and parental stress (estimated mean difference, −1.61 points; 95% CI, −3.24 to 0.02 points; *P* = .05).

**Table 3.** Raw Scores and Group Difference on All Secondary Outcomes at 1 and 3 Months

Measures	Raw Score, Mean (SD)						1 mo		3 mo	
	Control			Intervention			Estimated Mean Difference (95% CI)	*P* Value	Estimated Mean Difference (95% CI)	*P* Value
	Baseline	1 mo	3 mo	Baseline	1 mo	3 mo				
Parenting Sense of Competence Scale	71.8 (10.1)	70.8 (10.9)	71.2 (11.0)	69.6 (9.07)	72.4 (8.6)	73.5 (10.0)	3.33 (1.37 to 5.29)	.002	4.08 (1.96 to 6.20)	.002
Knowledge of										
Mental health	5.6 (1.3)	5.6 (1.3)	5.7 (1.2)	5.5 (1.2)	5.7 (1.2)	5.9 (1.1)	0.19 (−0.11 to 0.48)	.21	0.19 (−0.07 to 0.44)	.15
Help-seeking information	16.1 (2.7)	16.6 (2.4)	16.7 (2.2)	15.9 (2.7)	17.4 (2.0)	17.6 (1.9)	0.95 (0.44 to 1.45)	<.001	0.99 (0.49 to 1.50)	<.001
Parental Stress Scale	37.7 (7.4)	40.4 (7.8)	39.8 (8.9)	39.8 (8.7)	40.3 (7.7)	38.8 (8.1)	−1.61 (−3.24 to 0.02)	.05	−2.39 (−4.37 to −0.40)	.02
Parent-Adolescent Communication Scale	69.7 (9.9)	69.5 (9.6)	70.2 (10.0)	67.6 (8.9)	69.8 (8.5)	70.1 (9.5)	2.13 (0.64 to 3.62)	.005	2.21 (0.48 to 3.95)	.01

All significant group differences were maintained at 3-month follow-up, with the intervention group reporting better parent-related outcomes than the control group, including parental competence (estimated mean difference, 4.08 points; 95% CI, 1.96-6.20 points; *P* < .001), knowledge of help seeking (estimated mean difference, 0.99 points; 95% CI, 0.49-1.50 points; *P* < .001), and parent-adolescent communication (estimated mean difference, 2.21 points; 95% CI, 0.48-3.95 points; *P* = .01). Parents in the intervention group reported significantly less parental distress then the control group (estimated mean difference, −2.39 points; 95% CI, −4.37 to −0.40 points; *P* = .02). No significant difference was observed for knowledge of mental health (estimated mean difference, 0.19 points; 95% CI, −0.07 to 0.44 points; *P* = .15) at 3 months.

The effects of the intervention on all outcomes measured at both points were similar using linear mixed models on repeated measures. No significant differences were observed between ethnic groups, although the number of Māori and Pacific participants was small.

Most participants (90.3%) in the intervention group rated the program as somewhat to very useful. The majority (98.1%) reported reading all the text messages, with the remaining participants reading more than half of the text messages received. A total of 98.1% agreed that text messages were a good way to deliver information, with 90.3% reporting they would recommend the program to others. More than half (57.7%) of the participants thought the program was too short. Parents’ open-ended feedback is presented in Table 4.

**Table 4.** Themes and Sample Quotes From Parents’ Open-Ended Feedback on the Program

Theme	Sample Quotes
Overall program	
Positive and encouraging	“It really felt empowering and reminded me of things that were important. I think you get lost in your life and there is always so much to do so this was really lovely.”
Helpful	“I think all parents could benefit from this programme.”
Enjoyable	“I enjoyed this programme. I liked how encouraging some of the messages were and I really liked the texts which had rationale for the information given.”
	“It was so good and I was surprised how quickly the month finished. I looked forward to the daily messages.”
Delivery	
Acceptable	“I love the idea of using technology to get this programme/education out there in an easy, nonevasive, accessible, and user-friendly way. It's really positively programmed too.”
Longer duration of the program	“I would love this to continue for another couple of weeks.”
Content	
Incorporate more practical tips	“I looked forward to the daily messages. I loved being encouraged. More practical ideas would be great.”
Mixing the order of the text messages	“The content could be better distributed. The first part were all positive messages around how to parent a teen. While the remaining messages were all about depression. An important topic but, perhaps 2 to 3 positive messages followed by 1 to 2 on depression could be a better mix.”
Mental health information irrelevant to current needs	“Depression is one problem for teens but not the problem for my child so more focus on general behavior might be good.”
	“This was a really useful course, but for me the depression side of things weren't as useful as the other messages. Although I can see how they may be very helpful to some parents.”
Abbreviated text was difficult to read	“Text shorthand was not easy to read.”
Recommendations	
Text messages for the child	“A good idea to reach out to teens on their mobiles too since many have them now.”
Text messages for both parents	“Options for texting to both parents—I forwarded all the messages to my husband and we sometimes discussed how we could/are applying them with our teens.”
Option to text back	“Maybe a text back option so that if parents were having problems they could text back with the issue and be referred where they need to be referred too.”
Additional information via email	“An email service would be good too. Short, simple and to-the-point information should be in the email.”

## Discussion

Our program of daily text messages designed to increase parental competence and improve mental health literacy delivered over 1 month led to a measurable difference in parents’ sense of competence. To our knowledge, this is the first study that provides preliminary evidence-based support for the effectiveness of a parenting program for parents of adolescents delivered solely via text messages.

Consistent with other face-to-face and online parenting programs, our program was effective in enhancing and sustaining several parent-related outcomes.^10,11,30^ Parents who received the program reported significantly higher parental competence than parents in the control group. Parental competence has been demonstrated to be strongly associated with parenting function (ie, parental warmth and involvement).^31^ Parents with high self-efficacy are more likely to feel confident in adopting effective parenting skills and are therefore more likely to exert a positive influence on their adolescents, with a reduction in adolescents’ depressive symptoms being one of many optimal psychosocial outcomes reported.^31,32^ Lower levels of parental stress were reported in parents who received the intervention. Increased parental competence has been associated with decreased parental stress.^32,33^ Although no correlation analysis was conducted, it is likely that parents who received the intervention felt more competent in their parenting and therefore found everyday parenting less stressful.

Our program improved parents’ knowledge of help-seeking options, with sustained effects at 3 months. Having knowledge of how and where to seek information and treatment options is a crucial component of mental health literacy.^34^ The most common help-seeking barrier reported by parents is not knowing where to seek help.^35^ It is likely that with specific links and contacts delivered via text messages, parents were able to refer to the information when needed. Nonetheless, no group difference was found on mental health knowledge. Our sample of parents reported high scores at baseline on mental health knowledge, leaving little room for improvement. It is also possible that the amount of information conveyed by the intervention was not sufficient to produce significant improvement. Our program was 4 weeks and limited to 160 characters per text (90 characters if emojis were used); therefore, we were restricted on the number of text messages on mental health knowledge.

It should be noted that some parents found the mental health information irrelevant to their adolescent. Parents preferred more positive and practical tips on parent-adolescent communication. Our program was nevertheless effective in improving parent-adolescent communication. Most parents indicated they wanted the program to be longer. Future development of our program should evaluate adding different modules, examine dosage, and obtain adolescent data for evaluation.

Importantly, the novelty of delivering the program via text messages was well received, with participants rating the program favorably and stating that they would recommend it to others. Low attrition rates suggest that parents have an interest in receiving the text messages. Our program was far shorter than many existing parenting interventions. It required minimal time commitment and was conducted with no therapist support. Text-messaging interventions are intrinsically proactive and can be accessed by users at any time.^36^ Compared with other computerized modalities, text messaging does not require logging in or tunneling through web pages, thus reducing perceived barriers to engagement.^36^ This also contrasts with traditional services, which often require action or attendance by the participant before they can impart information. This is particularly important for parents, as time constraints are a barrier to participation in services.^16,35^ Our program has the potential to reach a wider, culturally diverse population, including those living in rural and remote communities or internationally.

Our brief intervention may also be useful for engaging parents to seek further services when needed. Studies have found that parents who attended brief programs had increased likelihood of seeking additional support services.^37^ By providing website links and resources to support services using the convenience of text message, our program may encourage parents to access information and services. There is a large group of parents in the general population that could potentially benefit from the information provided via our program. Not all parents require an intensive level of parenting support; providing general information on positive parenting strategies and adolescent development may be sufficient to improve parent-related outcomes, as shown in our study.

### Limitations

This study has some limitations. There was overrepresentation by highly educated mothers from intact families. Such sociodemographic characteristics are typical of participants in other parenting programs. Further research is required to determine whether the intervention would have similar effects for families with different characteristics. As mHealth parenting programs in New Zealand for culturally diverse populations are limited, our program can be culturally adapted and evaluated for effectiveness in future studies.

For reasons of parsimony, we only included 1 parent per household. In future research and outside the research context, it is possible for all caregivers to receive the program, which may enhance the impact of the program. This appears to be desirable as many of our participants reported sharing the text messages with other family members.

Although multiple method and informant data sources are ideal, all outcomes were obtained by parents’ self-report, raising the possibility of self-report bias. However, the values of ratings by parents should not be dismissed, given their unique knowledge about their own role as parents.^38^ This study lacked adolescent outcome data, thus restricting the evaluation of actual benefits of the intervention for adolescents. Also, only 1- and 3-month follow-up data were collected; therefore, long-term effects are unknown. Nonetheless, our findings can provide preliminary data and inform future efforts.

## Conclusions

mHealth interventions targeting parents remain relatively rare but may be a promising approach to supporting parents by widening reach, increasing population-level impact, and reducing inequalities due to lack of access to existing services.^12^ The MyTeen program appears to be an effective and feasible way to reach and support large number of parents. It can be widely deployed as an early intervention to increase parental competence, provide mental health and help-seeking knowledge, reduce parental stress, and improve parent-adolescent communication. Offering the program universally minimizes the stigma associated with accessing parenting support. While our program may not meet the need of parents and families who require more intensive intervention or replace face-to-face therapy, it offers a valuable prevention service. It may represent a less expensive option for service delivery. Future study to investigate the cost effectiveness of the program will inform the scalability, sustainability, and potential cost savings of the program.

## Notes

Supplement 1. **Trial Protocol**

Supplement 2. **eTable.** Intervention Content Including the Domains, Strategies and Sample Text-Messages

Supplement 3. **Data Sharing Statement**
