# Supplementary material for: Effect of MyTeen SMS-Based Mobile Intervention for Parents of Adolescents: A Randomized Clinical Trial
Source: JAMA Netw Open. 2019 Sep 11;2(9):e1911120. doi: 10.1001/jamanetworkopen.2019.11120 (PMC6739724; doi:10.1001/jamanetworkopen.2019.11120)
Supplement: Supplement 1. — Trial Protocol [file jamanetwopen-2-e1911120-s001.pdf]

Confidential
Not for Public Circulation or Reproduction

# Protocol

## MyTeen – Increasing competence and mental health literacy: A mobile-based intervention to support parents of teenagers

**Trial Registration Number: ACTRN12618000117213**

**Principal Investigator**
**Dr Joanna Ting Wai Chu**
**National Institute for Health Innovation, The University of Auckland**

National Institute for Health Innovation


**©National Institute for Health Innovation, The University of Auckland, 2018**

# MyTeen Study Co-ordinating Centre

***Postal address***
National Institute for Health Innovation
School of Population Health
The University of Auckland
Private Bag 92019
Auckland Mail Centre
Auckland 1142
New Zealand
Tel: 64 9 373 7999
Web: www.nihi.auckland.ac.nz

***Street address***
National Institute for Health Innovation
Level 4, School of Population Health
Tamaki Campus
The University of Auckland
261 Morrin Road
Glen Innes
Auckland 1072
New Zealand


**©National Institute for Health Innovation, The University of Auckland, 2018**

# Steering Group Committee Members

**Dr Joanna Chu**
Principal Investigator

National Institute for Health Innovation
University of Auckland
Private Bag 92019
Auckland 1142, New Zealand
Emergency no: 021 373 378
Email: jt.chu@auckland.ac.nz

**Assoc Prof Robyn Whittaker**
Co-Investigator

National Institute for Health Innovation
University of Auckland
Private Bag 92019
Auckland 1142, New Zealand
Fax: 64 9 373 1710
Emergency no: 021 968 029
Email: r.whittaker@auckland.ac.nz

**Professor Chris Bullen**
Co-Investigator, Director

National Institute for Health Innovation
University of Auckland
Private Bag 92019
Auckland 1142, New Zealand
Tel: 64 9 373 7599 ext. 84730
Fax: 64 9 373 1710
Emergency no: 64 9 522 2780
Mobile no. + 6421415267
Email: c.bullen@auckland.ac.nz

**Dr Yannan Jiang**
Senior Statistician

National Institute for Health Innovation
University of Auckland
Private Bag 92019
Auckland 1142, New Zealand
Tel: 64 9 923-4725
Email: y.jiang@auckland.ac.nz

**Dr Karolina Stasiak**
Advisor

Faculty of Medical and Health Science
University of Auckland
Private Bag 92019
Auckland 1142, New Zealand
Tel: 64 9 923 3890
Email: k.stasiak@auckland.ac.nz

**Dr Matt Shepherd**
Advisor

Faculty of Education and Social Work
University of Auckland
Private Bag 92019
Auckland 1142, New Zealand
Tel: 64 9 6238899 ext 46368
Email: m.shepherd@auckland.ac.nz


**©National Institute for Health Innovation, The University of Auckland, 2018**

# Study Management Committee Members

**Dr Joanna Chu**
Principal Investigator

National Institute for Health Innovation
University of Auckland
Private Bag 92019
Auckland 1142, New Zealand
Emergency no: 021 373 378
Email: jt.chu@auckland.ac.nz

**Angela Wadham**
Senior Project Manager

National Institute for Health Innovation
University of Auckland
Private Bag 92019
Auckland 1142, New Zealand
Tel: 64 9 923 2337
Email: a.wadham@auckland.ac.nz

**Dr Yannan Jiang**
Senior Statistician

National Institute for Health Innovation
University of Auckland
Private Bag 92019
Auckland 1142, New Zealand
Tel: 64 9 923-4725
Email: y.jiang@auckland.ac.nz

**Michelle Jenkins**
Lead data manager

National Institute for Health Innovation
University of Auckland
Private Bag 92019
Auckland 1142, New Zealand
Tel: 64 9 373 7599 ext. 84552
Email: m.jenkins@auckland.ac.nz

**Mahfuz Rahman**
Developer

National Institute for Health Innovation
University of Auckland
Private Bag 92019
Auckland 1142, New Zealand
Tel: 64 9 373 7599 ext. 84552
Email: m.rahman@auckland.ac.nz


**©National Institute for Health Innovation, The University of Auckland, 2018**

Confidential
Not for Public Circulation or Reproduction

# Study Centre

**National Institute for Health Innovation**

School of Population Health
University of Auckland
Private Bag 92019
Auckland 1142
New Zealand

# Co-ordinating Centre (NIHI) Staff

| Name | Position | Telephone 09 373 7599 ext | Email |
|---|---|---|---|
| **Dr Joanna Chu** | Research Fellow | | jt.chu@auckland.ac.nz |
| **Assoc Prof Robyn Whittaker** | Co-investigator | | r.whittaker@auckland.ac.nz |
| **Prof Chris Bullen** | Director | 84730 | c.bullen@auckland.ac.nz |
| **Dr Yannan Jiang** | Senior statistician | | y.jiang@auckland.ac.nz |
| **Angela Wadham** | Project manager | | a.wadham@auckland.ac.nz |
| **Michelle Jenkins** | Data manager | 84552 | m.jenkins@auckland.ac.nz |
| **Mahfuz Rahman** | Developer | | m.rahman@auckland.ac.nz |

# Project Sponsors

The principal financial sponsor of this trial is the National Science Challenge: A Better Start Cure Kids. [Project grant number: 3713711]. The design, conduct, analyses and interpretation of trial results will be made independent of the trial sponsor.


**©National Institute for Health Innovation, The University of Auckland, 2018**

Confidential
Not for Public Circulation or Reproduction

# Signature Page

**Author(s):**

| **Authors Name** | **Signature:** | **Date:** |
|---|---|---|
| Dr Joanna Chu | ____________________ | __________ |

| **NIHI Signatory: Director** | **Signature:** | **Date:** |
|---|---|---|
| Professor Chris Bullen | ____________________ | __________ |

| **Revision Chronology:** | Date | Type |
|---|---|---|
| MyTeen Study Protocol Version 1.0 | 30/01/2018 | Original |
| MyTeen Study Protocol Version 2.0 | 14/12/2018 | Amendment 1 |


**©National Institute for Health Innovation, The University of Auckland, 2018**

# Table of contents

**MYTEEN STUDY CO-ORDINATING CENTRE** .......... **2**
**STEERING GROUP COMMITTEE MEMBERS** .......... **3**
**STUDY MANAGEMENT COMMITTEE MEMBERS** .......... **4**
**STUDY CENTRE** .......... **5**
**CO-ORDINATING CENTRE (NIHI) STAFF** .......... **5**
**PROJECT SPONSORS** .......... **5**
**SIGNATURE PAGE** .......... **6**
**TABLE OF CONTENTS** .......... **6**
**1. OVERVIEW** .......... **9**
**2. STUDY PLAN SCHEMATIC** .......... **11**
2.1 Content Development .......... 11
2.2 Randomised Controlled Trial .......... 12
**3. BACKGROUND** .......... **13**
3.1 The need for depression prevention and early intervention .......... 13
3.2 Parents as an important target for prevention and early intervention .......... 13
3.3 Barriers to accessing services .......... 14
3.4 Mobile Health (mHealth) intervention in supporting parents of adolescents .......... 14
**4. RATIONALE FOR THE PRESENT STUDY** .......... **15**
**5. STUDY OBJECTIVES** .......... **15**
**6. STUDY DESIGN** .......... **16**
6.1 Content Development .......... 16
*6.1.1 Inclusion criteria .......... 16*
*6.1.2 Exclusion criteria .......... 16*
*6.1.3 Recruitment .......... 16*
*6.1.4 Study procedures .......... 16*
*6.1.5 Withdrawal criteria .......... 16*
6.2 Randomised Controlled Trial .......... 16
*6.2.1 Inclusion criteria .......... 16*
*6.2.2 Exclusion criteria .......... 17*
*6.2.3 Recruitment .......... 17*
*6.2.4 Study procedures .......... 17*
*6.2.5 Randomisation .......... 17*
*6.2.6 Blinding .......... 18*
*6.2.7 Study intervention .......... 18*
*6.2.8 Withdrawal criteria .......... 18*
*6.2.9 Baseline assessments .......... 18*
*6.2.10 Primary outcome measure .......... 18*
*6.2.11 Secondary outcome measures .......... 18*
*6.2.12 Schedule of intervention and follow-up .......... 20*
**7. STATISTICAL CONSIDERATIONS** .......... **20**
7.1 Sample size .......... 20
7.2 Data analyses .......... 21
7.3 Data management .......... 21
**8. ETHICAL APPROVAL AND CONSENT** .......... **22**
8.1 University of Auckland Human Participants Ethics Committee (UAHPEC) approval .......... 22
8.2 Informed consent .......... 22


**©National Institute for Health Innovation, The University of Auckland, 2018**

Confidential
Not for Public Circulation or Reproduction

**9. ASSESSMENT OF SAFETY / ADVERSE EVENT REPORTING ........ 22**
**10. RELEVANCE TO HEALTH ........ 22**
**11. DISSEMINATION OF RESULTS ........ 22**
11.1 Trial registration ........ 22
11.2 Study participants ........ 23
11.3 The general public ........ 23
11.4 Academic/professional colleagues ........ 23
11.5 Health service funders and providers ........ 23
11.6 Iwi/ Māori ........ 23
11.7 Pacific Island communities ........ 23
**12. ADMINISTRATIVE SECTION ........ 23**
12.1 Adherence to the protocol ........ 23
12.2 Protocol revision procedures ........ 23
12.3 Case report form procedures ........ 24
12.4 Monitoring/ Source document verification ........ 24
12.5 Data confidentiality and security ........ 24
12.6 Reporting schedule ........ 24
12.7 Record retention policy ........ 24
12.8 Insurance ........ 24
12.9 Ownership of data and publication policy ........ 24
12.10 Data Sharing ........ 25
**13. REFERENCES ........ 26**
**14. APPENDIX 1 – TERMS OF REFERENCE ........ 29**
14.1 Steering Group Committee ........ 29
14.2 Study Management Committee ........ 29
**15. APPENDIX 3 – PROPOSED TIMELINE ........ 30**
15.1 Trial Timeline ........ 30
15.2 Key milestones ........ 30
**16. APPENDIX 4 – SUMMARY OF PROTOCOL AMENDMENTS ........ 31**


**©National Institute for Health Innovation, The University of Auckland, 2018**

Confidential
Not for Public Circulation or Reproduction

# 1. Overview

**Title of study**: MyTeen – Increasing competence and mental health literacy: A mobile-based intervention to support parents of teenagers

**Investigators and study centres**
This study has been designed by independent investigators at the National Institute for Health Innovation (NIHI), School of Population Health, University of Auckland, Auckland. The overall design and conduct of this trial is the responsibility of the principal investigator and members of the Steering Committee and Study Management Committee. Publication of data from this trial will be the responsibility of members of the Steering Committee. The study will be co-ordinated from NIHI.

**Study period:** July 2017 – June 2019

**Objectives:** To design and evaluate the effectiveness of a SMS-based mobile intervention that promotes parental competence and mental health literacy for preventing adolescent mental health problems.

**Study design and methodology:** The research comprises two phases. In the content development phase, six focus groups will be conducted with parents of adolescents (10-15 years of age), with group size ranging from 6-8. Parents' needs, preferences and their input on the content of the intervention will be sought. In the evaluation phase, a two-arm randomised controlled trial (RCT) will be conducted to assess the effectiveness and acceptability of a SMS-based mobile intervention, compared with a care-as-usual control group.

**Study population:** Around 36-48 parents will be invited to take part in a focus group in the development phase. In the RCT, 214 parents will be randomised to either intervention or care as usual group (107 per arm).

**Main criteria for inclusion:** For the focus groups, parents will be eligible for inclusion in the study if they have a child aged between 10-15 years, are able to speak and understand English, able to attend the study site, and willing to provide consent. For the RCT, parents will be eligible for inclusion in the study if they indicate at screening that they have an adolescent child aged between 10-15 years of age; have access to a mobile phone; are not currently receiving any professional assistance for their own and/or child's mental health problems; possess adequate knowledge of the English language; and are willing to participate in the study and provide follow-up information at scheduled points of the study.

**Exclusion criteria:** Parents that do not meet the inclusion criteria will be excluded from the study. In addition, for the RCT, parents that show high level of stress as reported by the Parental Stress Index (i.e., ≥ 72) at screening will be excluded and directed to professional services.

**Criteria for evaluation**
*Primary outcome*

- Change in parental competence as measured by the Parental Sense of Competence Scale (PSOC) from baseline to 1-month follow up.


©National Institute for Health Innovation, The University of Auckland, 2018 

*Secondary outcomes*
Measured at one and three months post-randomisation:

- Knowledge of mental health issues in youth
- Continued improvement in parental competence (PSOC, 3-months follow up)
- Mental help seeking behaviour (Mental Health Literacy Scale, MHLS)
- Level of parental distress (Parental Stress Scale, PSS)
- Parent-adolescent communication (Parent-adolescent Communication Scale, PACS)
- Satisfaction with the programme

*Sample size*
214 participants (107 in each arm), will provide 80% power at p=0.05 to detect an effect size of a 2.5 difference in the Parenting Sense of Competence scale (PSOC) score at 1 month follow up (SD=5.8). This sample size estimate includes an estimated 20% loss to follow up in both trial arms.

**Analytic methods**
Qualitative analysis methods, drawing on the general inductive approach, will be used to identify topics and themes from the data collected by focus groups.

*Statistical analysis*: All analyses will be carried out on an intention-to-treat (ITT) basis. Sensitivity analyses will be undertaken to determine the impact of missing data.Generalised linear mixed model will be used to assess the overall intervention effect on each outcome at 1 and 3 months, adjusting for baseline outcome value and ethnicity (stratification factor). Repeated measures on the same participant will be taken into account in analysis using a random subject effect. Model-adjusted estimates of group difference and 95% confidence intervals will be reported with associated p-values. All statistical tests will be two-sided at 5% significance level. Subgroup analysis by ethnicity will be conducted to evaluate the consistency of intervention effects across ethnic groups, if the recruitment targets are met.

**Funding**
The National Science Challenge: A Better Start/Cure Kids. [Project grant number: 3713711] is the principal sponsor of this trial.


**©National Institute for Health Innovation, The University of Auckland, 2018**

# 2. Study Plan Schematic

## 2.1 Content Development

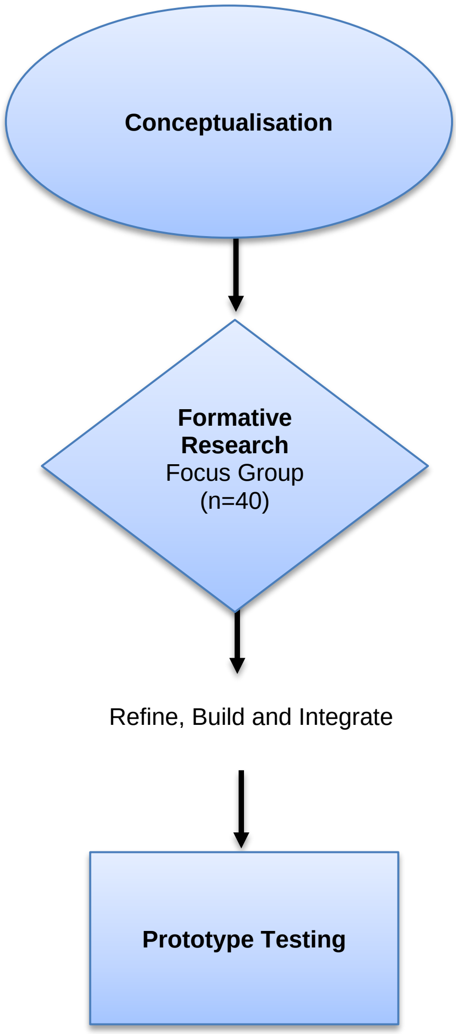



**©National Institute for Health Innovation, The University of Auckland, 2018**

Confidential
Not for Public Circulation or Reproduction

## 2.2 Randomised Controlled Trial

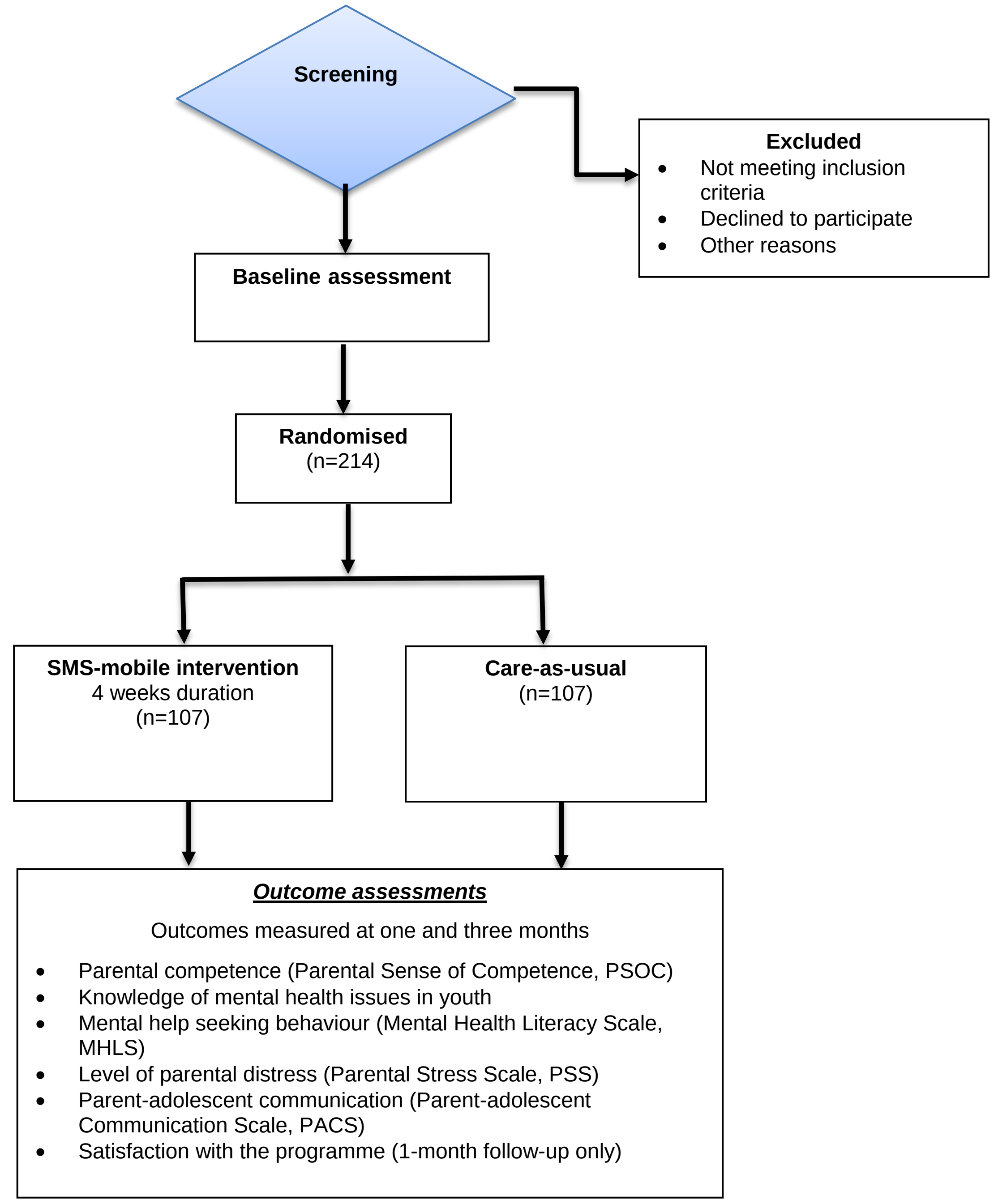



**©National Institute for Health Innovation, The University of Auckland, 2018**

# 3. Background

New Zealand has one of the highest rates of youth suicide in the developed world [1]. Depressive disorder is common, affecting at least a fifth of adolescents by the age of 18 [1]. Depression is a leading cause of morbidity in adolescents, and a major risk factor for suicide, the second most common cause of death in this age group [2]. Depression has a high rate of relapse and commonly starts in adolescence. The effect of depressive disorder is pervasive and affects not only function but overall development. Depressive disorder is associated with poor academic functioning, social dysfunction, substance use, attempted and completed suicide [3, 4]. Co-morbidity is high, with up to half of those with major depressive disorder having a life time occurrence of another psychiatric disorder [4]. In New Zealand, depressive disorder is a major health issue amongst adolescents, with prevalence rates of 4-8% at the age of 15 rising rapidly to 17-18% by the age of 18 [5]. Rates are higher for young people of Māori and Pacific: 23% of Māori girls, and 22% of Pacific girls report depression in the clinical range on the Reynolds Adolescent Depression Scale (RADS), compared with 15% of New Zealand European girls (odds ratio Māori 1.46 95% CI 1.20-1.79; Pacific 1.25 95% CI 0.96-1.63). Among Māori and Pacific boys the rates are higher than those for NZ European boys but this difference is not statistically significant [1]. High rates of depression are linked to teenage pregnancy, substance abuse, suicidal ideas and completed suicide.

## 3.1 The need for depression prevention and early intervention

Evidence from the past two decades suggests that prevention programmes reduce the incidence of mental health problems and the importance of preventive interventions has been emphasized by numerous expert panels [6, 7]. The serious developmental consequences of adolescent depression and the associated treatment challenges and the high costs once it has developed underscore the need for programmes aimed at prevention [8, 9]. Current clinical practice, generally limited to treating depression in its acute phase, fails to alleviate the disease burden in a significant way at the population level [10]. Two decades ago, Fergusson and colleagues [11] reported that up to 80% of young people with depression did not get treatment. More recent studies suggest that the majority of young people with depression still do not receive treatment [12]. Māori and Pacific youths' higher prevalence of mental illness is not matched by higher access to mental health services. Despite improvements in service provision over the last few decades, including the availability of some kaupapa Māori services, Māori tend to present to health services at a later stage and with more severe health problems than non-Māori [13, 14].

## 3.2 Parents as an important target for prevention and early intervention

A critical factor in an adolescent's outcome is the extent to which parents are responsive and supportive to their children's developmental needs and skilled in managing their children's behaviour [15, 16]. Research from the field of developmental psychopathology links a number of family risks and protective factors (e.g. quality of parent-child relationship, parental self-efficacy, parental adjustment, and parenting practices) to adverse mental health outcomes in adolescents [15, 17, 18]. Families with frequent arguments, escalating hostility, criticism, or anger create a stressful family environment that can undermine adolescents' coping resources and increase their risk for depression [3, 17, 18]. Parents can provide key resources that mediate risk for youth exposed to high levels of adversity [18, 19] and the protective role of positive parenting holds, irrespective of socio-economic status and levels of neighbourhood distress [20].

Help-seeking behaviours for depression are highly complex among adolescents in the general population [12, 21] as well as for Māori and Pacific youths [1, 13]. Parents are also more likely to overlook depression and other internalising disorders because they often manifest in less disruptive ways then externalising disorders [18]. If adolescent depression is to be prevented, recognised early and appropriate action taken, then a key strategy is to enhance positive parenting practices and increase parents' mental health literacy - the ability


**©National Institute for Health Innovation, The University of Auckland, 2018**

to recognise specific disorders; knowing how to seek mental health information; knowledge of risk factors and cause, of professional help available; and attitudes that promote recognition an appropriate help seeking [8, 17, 22]

Grounded in evidence-based approaches, including social learning models, self-regulation theory, and cognitive behavioural therapy, parenting programmes aimed at strengthening parenting skills and increasing knowledge on adolescent development have led to significant improvement in parent-adolescent relationships and a reduction in adolescent mental health problems [6, 23-27]. Studies have reported that parent's acknowledgement of their child's depression is associated with adolescent's readiness (i.e., perceptions about viewing depression as a problem, understanding the symptoms, and wanting to get help) to seek professional help [28]. Young people themselves also see parents as one of the most important sources of support for receiving help on mental health problems [29]. The prevention and early intervention efforts that effectively up skill parents thus have great potential in preventing depression in youths.

## 3.3 Barriers to accessing services

Even when promising programmes are available to support parents, engaging families can be challenging, with engagement rates as low as 10% for supplemental parenting training when added to individual treatment for depressed adolescents [30]. Traditional face-to-face intervention is resource intensive and, depending on the setting, can be difficult to implement on a large scale with limited reach to some population groups. Barriers to accessing services include perceived stigma, shame, cost, transport, waitlists, scepticism, distrust of the system/professionals, work commitments, rural isolation, and low mental health literacy, including poor awareness of signs/symptoms and resources. While home visits are known to be effective for reaching parents, there are limitations to resourcing and some families are also resistant to these [31].

For Māori and Pacific parents, lack of cultural support offered by health service and misinformed perceptions by medical practitioners may further contribute to the underutilisation of mental health services [4, 32]. Local research has found that in comparison to Pakeha parents, Māori and Pacific parents often feel that they do not have sufficient access to information needed for parenting [33]. This suggests the need for more effective strategies to engage Māori and Pacific parents in health care services that overcome such barriers. Sole reliance on traditional modes of intervention is likely to be insufficient for the level of need and demand. Innovative interventions that involve parents and that make use of technology-based platforms might be effective and efficient in preventing and mitigating adolescent mental health problems.

## 3.4 Mobile Health (mHealth) intervention in supporting parents of adolescents

MHealth interventions have great potential for public health impact because of their broad reach and convenience [34]. mHealth offers a wide range of potential benefits over traditional approaches, such as (1) programmes can be delivered anywhere at any time, and for extended periods, facilitating regular communication and behavioural maintenance; (2) support via messages or notifications are sent directly to people in a time-sensitive manner, which means the programme can be designed to fit in with the individual's lifestyle and provide prompts at the most appropriate times (3) programme are more proactive (initiated by the service) than traditional services, which often require action or attendance by the participant before they can impart information or provide support; (4) they are flexible, and can be personalised and tailored to specific cultural, age group, and health needs; (5) reach is increased because the barriers of face-to-face contact (such as time, cost and travel) are removed and; (6) disparities in access across the socio-economic status gradient are decreased due to the high penetration of mobile phones across these groups [34]. The ease, accessibility, and autonomy-focused characteristics of mHealth interventions offer promise in


**©National Institute for Health Innovation, The University of Auckland, 2018** **Date: 14/12/18**

supporting parents to prevent and mitigate mental health problems in their children. Opinion surveys indicate that parents who are interested in family programmes have a stronger preference to receive such support through technological platforms over face-to-face delivery [35, 36].

While text messaging may not be considered a 'novel' mobile phone application, globally, it remains the most widely used. It is also inexpensive to develop and deliver and it requires minimal technological 'know how'. Text messaging also requires very basic, low-cost phones (to receive and send messages), which reduces potential socioeconomic disparity of access ('digital divide'). Text messaging programmes have successfully promoted parenting behaviour change in a number of important domains for parents of young children: decreasing the likelihood of abuse and neglect, increasing childhood vaccinations, and encouraging healthy pregnancies [37-39]. Studies have reported that text messaging interventions were well received by parents of various populations including those that are socially deprived [39]. Locally, our text-messaging intervention programme doubled smoking cessation rates and was as effective in Māori as non-Māori [40, 41]. That research formed the basis of the national text messaging support programme now available via Quitline.

Mobile phone use is very common amongst Māori and Pacific groups, more so than Pakeha. In a survey conducted in 2014, smartphone ownership in New Zealand was more common amongst Māori and Pacific people (70%) than Europeans (55%), and Māori and Pacific users were more likely to report current use of a smartphone than a year ago (59%) compared to Europeans (46%) [42]. This situation presents, therefore an excellent opportunity to deliver a widely accessible intervention to support Māori and Pacific parents in preventing adolescent mental health problems. The simple and brief nature of text-messages may appeal to Māori and Pacific parents as the approach is more personal, private, and less-confrontational then traditional face-to-face services. While there is evidence to suggest the feasibility and effectiveness of SMS mobile-based interventions to address health issues [37, 38, 43], its application and impact among adolescent parent populations are unknown. We hypothesise that a SMS-based mobile intervention will have great potential to reach and support parents of adolescents.

## 4. Rationale for the Present Study

The prevalence of mental health problems in youth is substantial; and efforts aimed at strengthening parenting skills and increasing knowledge on adolescent development hold much promise to prevent and mitigate adolescent mental health problems. To date there have been no reported investigations on the efficacy of delivering a parenting support intervention for parents of adolescents via a mobile-based intervention. We propose a two stage project to design and evaluate the effectiveness of a SMS-based mobile intervention for parents of adolescents on promoting parental competence and mental health literacy.

## 5. Study Objectives

To design and evaluate the effectiveness of a SMS-based mobile intervention that promotes parental competence and mental health literacy in for preventing adolescent mental health problems. Our primary hypothesis is that compared to the control group, parents receiving the SMS-based mobile intervention would report higher levels of competence in managing mental health issues in youth at 1 month follow up. Secondary hypotheses include improvements in the following outcomes at 1 and 3-months follow up: (a) knowledge of mental health issues in youth; (b) mental health help seeking behaviour; (c) levels of parental distress; (d) parent-adolescent communication; and (e) satisfaction with the programme.


**©National Institute for Health Innovation, The University of Auckland, 2018**

# 6. Study Design

## 6.1 Content Development (Focus Groups)

The aim is to develop a SMS-based mobile intervention that is culturally relevant and acceptable to parents of adolescents. Focus groups will be conducted to understand the needs and preferences of parents, and to seek their input on the content of the intervention.

### *6.1.1 Inclusion criteria*

Parents will be eligible for inclusion in the study if they have a child aged between 10-15 years, are able to speak and understand English, able to attend the study site, and willing to provide consent.

### *6.1.2 Exclusion criteria*

Parents that do not meet the inclusion criteria will be excluded from the study.

### *6.1.3 Recruitment*

Parents will be recruited via social media advertisement (e.g., facebook), advertisement about the study in local newspapers, word of mouth, and flyers distributed in community centres and schools.

### *6.1.4 Study procedures*

Potential participants can either call a phone number to speak with a research assistant or leave their contact details via email. The research assistant will contact the participant and explain the study and ask screening questions to ascertain their eligibility to participant. Those who meet the study inclusion criteria will be sent a participant information sheet and an appointment letter. Written consent will be obtained at the study site on the day of the focus group. Focus groups will last approximately 90 minutes, including completion of a brief questionnaire collecting demographic information. An interview guide with prompts will be used to guide the focus group sessions. Sessions will be audio-recorded with the consent of the participants. Refreshments will be provided and each participant will receive a $50 voucher in appreciation of his or her time and cost of transport to and from the study centre.

### *6.1.5 Withdrawal criteria*

As part of the informed consent procedure and in accordance with best practice guidelines, the participant information and consent processes will clearly state that participation is voluntary and parents will be free to withdraw at any stage of the research. Other reasons for withdrawal include the study being terminated for any reason. However, they will not be able to withdraw the data from the focus group discussions.

## 6.2 Randomised Controlled Trial

The aim is to evaluate the effectiveness and acceptability of the SMS-based mobile intervention, compared with a care-as-usual control group.

### *6.2.1 Inclusion criteria*

Parents will be eligible for inclusion in the study if they indicate at screening that they have an adolescent child aged between 10-15 years of age; have access to a mobile phone; are not currently receiving any professional assistance for their own and/or child's mental health problems; possess adequate knowledge of the English language; and are willing to participate in the study and provide follow-up information at scheduled points of the study. Only one parent from two-parent households will be invited to participate.


**©National Institute for Health Innovation, The University of Auckland, 2018**

#### *6.2.2 Exclusion criteria*

Parents that do not meet the inclusion criteria will be excluded from the study. Parents that show high level of stress as reported by the Parental Stress Index (i.e., ≥ 72) at screening will be excluded and directed to professional services.

#### *6.2.3 Recruitment*

The SMS-based mobile intervention is intended to serve a diverse population of parents in the community. Specific recruitment methods will include:

- Using social media e.g., Facebook, Google Ads
- Using posters, flyers, and other advertising material
- Word of mouth
- Invitation through schools, community organisations such as sports and cultural clubs (e.g., kapa haka, waka ama), faith communities (churches, mosques, temples) and marae.

#### *6.2.4 Study procedures*

Potential participants can either call or text a phone number to speak with a research assistant or leave their contact details via email. The research assistant will contact the participant and explain the study, obtain verbal consent to ask screening questions to ascertain their eligibility -for the study.

Eligible participants who indicate interest will be sent an email with the participant information sheet and consent form for them to read. A separate email will be sent from the study database containing a link to the first online questionnaire. Before they complete the questionnaire they will be asked to provide e-consent.

Participants will then be directed to complete the baseline assessment.

Randomisation will be performed upon completion of the baseline assessment, and participants will be notified via email which group they have been allocated to. Those that are randomised into the intervention group will receive the intervention programme the day following the notification email. Participants that are randomised into the care-as-usual group will receive no intervention from the research group.

At one-month post randomisation, participants in both groups will receive an email directing them to complete the 1-month follow up assessment. Another email will be sent at 3-months for participants to complete the 3-months follow up assessment. Reminder emails and/or texts will be sent if participants do not complete the assessments. A follow-up phone call will be made to the participant if the online assessment is not completed after two reminders.

Upon completion of the 3-months follow up, the SMS-based mobile intervention will be offered to participants in the control group. Each participant will receive a $20 petrol voucher in appreciation of his or her time given to the study.

#### *6.2.5 Randomisation*

Randomisation will be performed at the individual level. Participants (N=214) that fulfil entry criteria and have completed baseline assessment will be randomised at a 1:1 ratio to either a SMS-based mobile intervention group or to a care-as-usual ‘control’ group. The randomisation sequence will be generated by the trial statistician using block randomisation with variable block sizes of 2 or 4, and stratified by Māori, Pacific and non-Māori/non-Pacific. The final randomisation lists will be concealed in the database until the point of randomisation.


**©National Institute for Health Innovation, The University of Auckland, 2018**

#### *6.2.6 Blinding*

Due to the nature of the intervention, it will not be possible to blind participants or research staff to the different conditions.

#### *6.2.7 Study intervention*

Intervention: All intervention participants will receive a tailored programme of text messages (SMS) via their mobile phone. The messages will provide instructional, informational, and emotional support. These will include evidence-based information on the nature and symptoms of depression, understanding treatment options, strategies to improve parent-child communication, parent self-care, and useful links to resources. The intervention will be delivered over 4 weeks, and the details of the content, frequency, and intensity of the intervention will be developed and fine-tuned in the developmental phase of the study. Participants will receive all the text-messages free of charge.

Care as usual: Participants allocated to the care-as-usual control group will receive no intervention from the research team, and can access alternative services if they so desire. On completion of the 3-months follow up assessment, participants will be offered the SMS mobile-based intervention programme.

#### *6.2.8 Withdrawal criteria*

As part of the informed consent procedure and in accordance with best practice guidelines, the participant information and consent processes will clearly state that participation is voluntary and parents will be free to withdraw at any stage of the research. Other reason for withdrawal include the study being terminated for any reason.

#### *6.2.9 Baseline assessments*

At the baseline assessment, the following data will be collected:

- **Demographic data**: age, sex, marital status, ethnicity, education level, employment status, household income, family structure, child's age, child's sex, and child's ethnicity.

#### *6.2.10 Primary outcome measure*

The Parental Sense of Competence (PSOC) measures parental self-esteem on two dimensions: Satisfaction and Efficacy. Satisfaction section examines the parents' anxiety, motivation and frustration, while the Efficacy section looks at the parents' competence capability levels and problem-solving abilities in their parental role. The constructs of satisfaction and efficacy are closely linked with a host of positive family interactions as well as with positive child development. The total score of PSOC is calculated as the sum of 17 items, and has a possible range of 17 to 102.The PSOC appears to be sensitive to changes resulting from brief parenting support. In a New Zealand sample, the scale have good internal reliabilities of 0.81 and 0.88 for the satisfaction and efficacy subscales, respectively.

#### *6.2.11 Secondary outcome measures*

The following secondary outcome measures will be assessed:

- **Knowledge of mental health issues in youth (Fox et al., 2012) -** Parent knowledge of depression will be measured by a 7-item scale. Participants will be asked to rate the extent to which they agree or disagree with the following: (a) depression can cause serious academic difficulties, (b) untreated depression can result in death by suicide, (c) youth with depression are likely to be violent towards others, (d) teens with depression usually get better on their own, (e) depression is caused by bad or weak character, (f) there are effective treatments available for teens with depression, and (g) asking teens if they think about suicide will cause them to become suicidal. The response categories for each item: "strongly agree," "somewhat agree," "somewhat disagree," "strongly disagree,"


**©National Institute for Health Innovation, The University of Auckland, 2018**

and “don’t know.” For items a, b, and f, a response of “strongly agree” or “somewhat agree” are considered correct. For items c, d, e, and g, a response of “strongly disagree” or “somewhat disagree” was considered correct. For all items, a response of “don’t know” was considered incorrect. The responses from each item are recoded such that each correct response gives a score of 1 and each incorrect response was given a score of 0. The scores are then summed to create the parent knowledge score which ranged from 0 to 7, where a higher score indicated greater knowledge of depression.

- **Continued improvement in parental competence (PSOC, 3-months follow up)**
- **Knowledge of where to seek mental health information (Mental Health Literacy Scale, MHLS, O’Connor & Casey, 2015)** Subscale on knowledge of where to seek information from the Mental Health Literacy Scale will be used. The subscale consists of 4-items, rated on a 5-point scale, ranging from *strongly disagree* to *strongly agree.* The scale has demonstrated good internal and test-retest reliability, and scores are significantly correlated with help seeking intentions.
- **Level of parental distress (Parental Stress Scale, PSS, Berry & Jones, 1995)** measures the levels of stress experienced by parents, taking into account of the positive and negative aspects of parenting. The scale consist of 18 items and are rated on a 5-point scale from ‘strongly disagree’ to ‘strongly agree’ to generate a total score. The scale has good internal reliability (.83), and test-retest reliability (.81).
- **Parent-adolescent communication (Parent-adolescent Communication Scale, PACS, Olson & Barnes, 1982)** measures the quality of communication between parent and adolescent. The scale consist of 20 items and are rated on a 5-point scale from ‘strongly disagree’ to ‘strongly agree’ to generate a total score and two subscale score (open family communication and problems in family communication). The scale have good internal reliabilities for both sub scales (0.87 and 0.78, respectively) and test-retest reliabilities (0.78 and 0.77, respectively).
- **Satisfaction with the programme:** Program satisfaction is measured by five items, (a) “How much did you like this program?”,(b) “Did the program meet your expectations?”, (c) “Would you recommend this program to your friends and relatives?” Each item is rated on a five-point Likert scale ranging from *strongly disagree* to *strongly agree.*
- **Qualitative data:** Exit interviews will be done with a subset of participants to capture participants’ experience with the programme.


**©National Institute for Health Innovation, The University of Auckland, 2018**

*6.2.12 Schedule of intervention and follow-up*

Outcome assessments will be measured at baseline, then one and three months post-randomisation [Table 1].

**Table 1: Details of follow-up**

| Timing | | 0 Week | 1 month | 3 months |
|---|---|---|---|---|
| Description | Screening | Baseline data collection + Randomisation | Follow-up data collection | Follow-up data collection |
| CRF | | | | |
| **General data** | | | | |
| Verbal informed consent | ✓ | | | |
| E-consent | | ✓ | | |
| Eligibility | ✓ | | | |
| Randomisation | | ✓ | | |
| Age, sex, ethnicity | | ✓ | | |
| Socioeconomic position | | ✓ | | |
| Family structure | | ✓ | | |
| Child information | | ✓ | | |
| Contact details | ✓ | | ✓ | ✓ |
| **Primary Outcome** | | | | |
| Parental Competence (PSOC) | | ✓ | ✓ | ✓ |
| **Secondary Outcome** | | | | |
| Knowledge of mental health issue (Parent knowledge of depression) | | ✓ | ✓ | ✓ |
| Mental health help seeking | | ✓ | ✓ | ✓ |
| Parental Distress (PSS) | ✓ | | ✓ | ✓ |
| Parent-adolescent communication (PASC) | | ✓ | ✓ | ✓ |
| Programme satisfaction (Intervention Only) | | | ✓ | |
| **Exit Interview** | | | | |
| Feedback | | | ✓ | |

# 7. Statistical Considerations

## 7.1 Sample size

For the focus groups, we aim to recruit 40 parents (30% Māori, 30% Pacific). Six focus groups (6-8 parents per group) are proposed. However, the number of groups may decrease if the researcher feel that data saturation is reached. This will be determined by debriefing and reviewing notes following each focus group session.

For the RCT, we aim to recruit 214 parents in total (one parent per household; 30% Māori, 30% Pacific). This sample size (107 in each arm), will provide 80% power at p=0.05 to detect an effect size of a 2.5 difference in the Parenting Sense of Competence scale (PSOC) score at 1 month follow up (SD=5.8). This sample size estimate includes an estimated 20% loss to follow up in both trial arms.


**©National Institute for Health Innovation, The University of Auckland, 2018**

## 7.2 Data analyses

All focus groups will be conducted by a trained qualitative researcher and will be recorded (with permission) and transcribed verbatim. A general inductive approach will be followed, which allows research findings to emerge from multiple readings of the raw data. NVivo9 software will be used to manage the transcripts and facilitate the analysis process, and to identify themes and categories.

Data from the RCT will be entered into a RedCap database, and following cleaning and datalock, extracted into SAS (version 9.4) for analysis. All data analyses will be specified *a priori* in a statistical analysis plan. No interim analysis will be undertaken. *Baseline characteristics:* Baseline data collected from all participants will be summarised by treatment group, overall and by ethnicity (Māori, Pasifika and non- Māori non-Pacific). Continuous variables will be presented as numbers observed, means and standard deviations. Categorical variables will be presented as frequencies and percentages. Since any differences between randomised groups at baseline could only have occurred by chance, no formal significance testing will be conducted. *Intervention effects:* Analysis will be carried out on an intention-to-treat basis including all randomised participants. Sensitivity analyses will be undertaken to determine the impact of missing data (if any) under different assumptions. Primary and secondary outcomes will be first summarised descriptively by treatment group at each time point. Generalised linear mixed model will be used to assess the overall intervention effect on each outcome at 1 and 3 months, adjusting for baseline outcome value and ethnicity (stratification factor). Repeated measures on the same participant will be taken into account in analysis using a random subject effect. Model-adjusted estimates of group difference and 95% confidence intervals will be reported with associated p-values. All statistical tests will be two-sided at 5% significance level. Subgroup analysis by ethnicity will be conducted to evaluate the consistency of intervention effects across ethnic groups, if the recruitment targets are met.

## 7.3 Data management

Information about study subjects will be kept confidential in keeping with the obligations set out in the Privacy Act 1993 and the Health Information Privacy Code 1994.

NIHI stores data either on The University of Auckland owned storage and servers, or on cloud services operated by a vendor with whom The University of Auckland have a contractual relationship. Data stored on The University of Auckland storage and servers will be managed in accordance with appropriate NZ Information Security Manual (NZISM) guidelines and relevant legislation including the Privacy Act 1993. Data stored using cloud services is maintained by the vendor and their security is assessed by 1). Relevant vendor certification or accreditations, 2). Independent audits of services conducted by 3rd parties, and 3). University of Auckland performing audits to test the vendor services. All data including voice recordings, transcripts, forms will be held securely at NIHI. All electronic data will be password protected and stored on the internet data management system (See Manual of Procedures).

Access to all study data will be restricted to research staff directly involved in conducting or monitoring the study. Confidentiality will be protected by the use of study registration numbers, and only aggregated and anonymous data will be reported. Personal information will be kept confidential and stored securely. Computerised information will be password protected and hard copy information kept in a locked filing cabinet. All reports from the study will be written in a way such that no individuals can be identified.


**©National Institute for Health Innovation, The University of Auckland, 2018**

# 8. Ethical Approval and Consent

## 8.1 University of Auckland Human Participants Ethics Committee (UAHPEC) approval

Ethics approval was obtained from the University of Auckland Human Participants Ethics Committee (UAHPEC). Reference number 019659, 22 September 2017.

## 8.2 Informed consent

Maintenance of confidentiality and compliance with the Privacy Act will be emphasised to all study participants. Participation in the study will be entirely voluntary. For the focus groups, participant information sheets and consent forms will be provided to participants at the time of the focus group. Participants will be able to ask questions and written consent will be obtained prior to the start of focus groups. For the RCT, verbal-consent will be obtained at the time of screening and registration. E-consent will be obtained once participants have had the opportunity to read the Participant Information Sheet and ask any questions to the members of the study team prior to completing the baseline assessment.

# 9. Assessment of Safety / Adverse Event Reporting

No adverse or serious adverse events are anticipated and thus these data will not be collected in this trial.

# 10. Relevance to Health

The proposal will generate knowledge with potential about a mHealth model of parenting support to enhance the effectiveness and impact of current mental health services. If effective, the intervention can be easily scaled-up for national roll-out, and be adapted to enhance support for a diverse parenting population (e.g., parents of young children, parents of older adolescents, and various ethnic groups) on mental health issues. Our research will contribute to the goal of improving outcomes for families and youths by 1) increasing parental competence and mental health literacy to promote early recognition and appropriate help seeking behaviours; 2) providing a low-cost, sustainable parenting intervention with broad population reach; and 3) using technology to reduce disparities in utilisation of existing services, as mobile phones are used by all regardless of socioeconomic status or ethnicity. Demand for mental health services in New Zealand far exceeds the current services offered, and new approaches are therefore needed to enhance access to support parents of adolescents. We will be able to provide new knowledge about the uptake and adherence to this type of intervention for parents of adolescents.

# 11. Dissemination of Results

A Knowledge-Transfer Exchange Strategy will ensure the findings have the greatest possible impact on parents and adolescents. The strategy will focus on key messages, target audiences, appropriate communication channels, activities and timing, and measures of success. NIHI has standard operating procedures in place which cover all aspects of research dissemination. Furthermore, a key strategic goal of NIHI is to "increase research impact" via increasing the number and impact of research outputs.

## 11.1 Trial registration

The trial will be registered online on the Australian New Zealand Clinical Trials Registry (ANZCTR).


**©National Institute for Health Innovation, The University of Auckland, 2018**

### 11.2 Study participants

Study participants will be informed about the trial results by being sent a plain language summary of the results - after the publication of the study results.

### 11.3 The general public

The general public will be informed about the trial via posting of the research findings on the University's and other relevant websites, both national and international. Opportunities to make presentations to local, national and international audiences will be actively pursued. Another dissemination pathway is media releases (national and international) at the time of journal publication.

### 11.4 Academic/professional colleagues

Academic/professional colleagues will be informed about the trial via publication in high impact, leading international journals. Less formal feedback will be given via the investigators' participation in the national and international research community. Opportunities to make presentations to local, national and international audiences will be actively pursued.

### 11.5 Health service funders and providers

Academic papers and summary reports will be provided to funders.

### 11.6 Iwi/ Māori

Dissemination of findings to Māori organisations, media and community groups as guided by Dr Matt Sheppard and members of the NIHI Māori Research Advisory Group.

### 11.7 Pacific Island communities

Dissemination of findings to Pacific organisations, media and community groups as guided by Dr Vili Nosa, Senior Lecturer, Head of Pacific Health Section, School of Population Health, University of Auckland.

## 12. Administrative Section

### 12.1 Adherence to the protocol

Except for a change that is intended to eliminate an immediate hazard to participants, the approved protocol will be conducted as described. Any significant protocol deviation will be documented.

### 12.2 Protocol revision procedures

All revisions will be discussed with, and approved by, the Study Steering Committee. If the revision is an "administrative letter", the principal investigator will submit it to the University of Auckland Human Participants Ethics Committee for their information. If the revision is an "amendment", the principal investigator will sign it. The principal investigator will submit the amendment to the University of Auckland Human Participants Ethics Committee for review and approval or favourable opinion prior to implementation. Documentation of approval signed by the chairperson or designee of the University of Auckland Human Participants Ethics Committee will be sent to the principal investigator.

If an amendment substantially alters the study design or increases the potential risk to the subject:


**©National Institute for Health Innovation, The University of Auckland, 2018**

- the consent form will be revised and submitted to the University of Auckland Human Participants Ethics Committee for review and approval or favourable opinion;
- participants currently enrolled in the study, if they are affected by the amendment, will be contacted by telephone and the amendment discussed and verbal consent re-obtained;
- the revised consent form will be posted to participants currently enrolled in the study if they are affected by the amendment;

## 12.3 Case report form procedures

All questionnaire information will be entered onto the electronic forms on the study website.

## 12.4 Monitoring/ Source document verification

No formal monitoring will take place for this study as it is deemed to be low-risk, all data is self-reported and entered directly into REDCap and participants give e-consent.

Central monitoring will occur with logic checks built into the database to check for data inconsistencies and missing data and data will be checked at regular intervals during the study for completeness and accuracy

## 12.5 Data confidentiality and security

All data including voice recordings, transcripts, forms will be held securely at NIHI. All electronic data will be password protected and stored on the internet data management system (See Manual of Procedures).

## 12.6 Reporting schedule

The principal investigator will provide annual reports of the progress, or completion, termination or discontinuation of the study to the University of Auckland Human Participants Ethics Committee (UAHPEC) and to the funder of this study.

## 12.7 Record retention policy

NIHI will retain study documents for 6 years from data lock). Staff involved in the trial will not destroy any records associated with the trial, without the prior approval of the principal investigator. If the principal investigator or any co-investigators withdraw from the study (e.g. relocation, retirement), any records they hold will be transferred to a mutually agreed upon designee (e.g. another co-investigator). Notice of such transfer will be given in writing to the Director of NIHI.

## 12.8 Insurance

Participants may be entitled to compensation from the Accident Compensation Corporation (ACC) for personal injury suffered as a result of treatment given as part of the trial (section 32 (4) of the Injury, Prevention, Rehabilitation and Compensation Act 2001 and section 13 of the Injury, Prevention, Rehabilitation and Compensation Amendment Act (No 2) 2005).

## 12.9 Ownership of data and publication policy

Individual study data will remain the property of individual study participants. NIHI will have the responsibility for storage, protection and retrieval of study data. The Steering Committee will have the responsibility for the safe guardianship and use of the data.

All publications will be approved by members of the Steering Committee. Study participants, the research assistants, members of the Management Committee who are not part of the Steering Committee, and study sponsors will be acknowledged in the final report and in all publications and presentations resulting from this trial.


**©National Institute for Health Innovation, The University of Auckland, 2018**

Confidential
Not for Public Circulation or Reproduction

### 12.10 Data Sharing

All requests for de-identified individual participant data or study documents will be considered, after publication of the results, where the proposed use aligns with public good purposes, does not conflict with other requests, or planned use by the Study Steering Committee, and the requestor is willing to sign a data access agreement. Contact will be via the corresponding author.


©National Institute for Health Innovation, The University of Auckland, 2018

# 13. References


1. Clark, T., et al., *Youth'12 Prevalence Tables: The health and wellbeing of New Zealand secondary school students in 2012*. 2013.
2. Kessler, R.C., et al., *Lifetime prevalence and age-of-onset distributions of mental disorders in the World Health Organization's World Mental Health Survey Initiative.* 2007.
3. Beautrais, A.L., P.R. Joyce, and R.T. Mulder, *Risk factors for serious suicide attempts among youths aged 13 through 24 years.* Journal of the American Academy of Child & Adolescent Psychiatry, 1996. **35**(9): p. 1174-1182.
4. Cummings, C.M., N.E. Caporino, and P.C. Kendall, *Comorbidity of anxiety and depression in children and adolescents: 20 years after.* Psychological bulletin, 2014. **140**(3): p. 816.
5. McLeod, G., L. Horwood, and D. Fergusson, *Adolescent depression, adult mental health and psychosocial outcomes at 30 and 35 years.* Psychological medicine, 2016. **46**(07): p. 1401-1412.
6. Commission, M.H., *Family Inclusion in Mental Health and Addiction Services for Children and Young People*. 2009, Mental Health Commission: Wellington.
7. Medicine, N.R.C.a.I.o., *Preventing mental, emotional, and behaviorual disorders among young people: Progress and possibilities*. 2009, National Academic Press: Washington, DC.
8. Garber, J., *Depression in children and adolescents: linking risk research and prevention.* American journal of preventive medicine, 2006. **31**(6): p. 104-125.
9. Horowitz, J.L. and J. Garber, *The prevention of depressive symptoms in children and adolescents: A meta-analytic review*. 2006, American Psychological Association.
10. Chisholm, D., et al., *Reducing the global burden of depression.* The British Journal of Psychiatry, 2004. **184**(5): p. 393-403.
11. Tyrer, P., et al., *The Nottingham study of neurotic disorder. Effect of personality status on response to drug treatment, cognitive therapy and self-help over two years.* The British Journal of Psychiatry, 1993. **162**(2): p. 219-226.
12. Zachrisson, H.D., K. Rödje, and A. Mykletun, *Utilization of health services in relation to mental health problems in adolescents: a population based survey.* BMC Public Health, 2006. **6**(1): p. 34.
13. Fleming, T.M., R.S. Dixon, and S.N. Merry, *'It's mean!'The views of young people alienated from mainstream education on depression, help seeking and computerised therapy.* Advances in Mental Health, 2012. **10**(2): p. 195-203.
14. King, M., A. Smith, and M. Gracey, *Indigenous health part 2: the underlying causes of the health gap.* The Lancet, 2009. **374**(9683): p. 76-85.
15. Steinberg, L., *We know some things: Parent–adolescent relationships in retrospect and prospect.* Journal of research on adolescence, 2001. **11**(1): p. 1-19.
16. Trudeau, L., et al., *Internalizing symptoms: Effects of a preventive intervention on developmental pathways from early adolescence to young adulthood.* Journal of youth and adolescence, 2012. **41**(6): p. 788-801.
17. Restifo, K. and S. Bögels, *Family processes in the development of youth depression: Translating the evidence to treatment.* Clinical psychology review, 2009. **29**(4): p. 294-316.
18. Yap, M.B.H., et al., *Parental factors associated with depression and anxiety in young people: A systematic review and meta-analysis.* Journal of Affective Disorders, 2014. **156**: p. 8-23.
19. Berzin, S.C., *Vulnerability in the transition to adulthood: Defining risk based on youth profiles.* Children and Youth Services Review, 2010. **32**(4): p. 487-495.



**©National Institute for Health Innovation, The University of Auckland, 2018**

20. Brody, G.H. and X. Ge, *Linking parenting processes and self-regulation to psychological functioning and alcohol use during early adolescence.* Journal of Family Psychology, 2001. **15**(1): p. 82.
21. Lee, J., et al., *Youth's help-seeking intentions for ADHD and depression: Findings from a national survey.* Journal of Child and Family Studies, 2014. **23**(1): p. 144-156.
22. King, K.A. and R.A. Vidourek, *Teen depression and suicide: Effective prevention and intervention strategies.* The Prevention Researcher, 2012. **19**(4): p. 15-18.
23. Chand, N., et al., *Promoting positive youth development: Through a brief parenting intervention program.* Youth Studies Australia, 2013. **32**(1): p. 29.
24. Chu, J.T.W., et al., *Parent and adolescent effects of a universal group program for the parenting of adolescents.* Prevention Science, 2015. **16**(4): p. 609-620.
25. Kacir, C.D. and D.A. Gordon, *Parenting adolescents wisely: The effectiveness of an interactive videodisk parent training program in Appalachia.* Child & Family Behavior Therapy, 2000. **21**(4): p. 1-22.
26. Prinz, R., *Dissemination of a multilevel evidence-based system of parenting interventions with broad application to child welfare populations.* Child welfare, 2009. **88**(1): p. 127.
27. Ralph, A. and M.R. Sanders, *Preliminary evaluation of the Group Teen Triple P program for parents of teenagers making the transition to high school.* Australian e-Journal for the Advancement of Mental Health, 2003. **2**(3): p. 169-178.
28. Rickwood, D.J., K.R. Mazzer, and N.R. Telford, *Social influences on seeking help from mental health services, in-person and online, during adolescence and young adulthood.* BMC psychiatry, 2015. **15**(1): p. 40.
29. Swords, L., E. Hennessy, and C. Heary, *Adolescents' beliefs about sources of help for ADHD and depression.* Journal of Adolescence, 2011. **34**(3): p. 485-492.
30. Shochet, I.M., et al., *The efficacy of a universal school-based program to prevent adolescent depression.* Journal of clinical child psychology, 2001. **30**(3): p. 303-315.
31. Azzi-Lessing, L., *Home visitation programs: Critical issues and future directions.* Early Childhood Research Quarterly, 2011. **26**(4): p. 387-398.
32. Strickett, E.J. and N.P. o te Maramatanga Internship, *Marginalising Maori adolescent parents.* 2012.
33. Reid, P. and B. Robson, *The state of Maori health.* State of the Maori nation: Twenty-first-century issues in Aotearoa. Auckland: Reed, 2006.
34. Free, C., et al., *The effectiveness of M-health technologies for improving health and health services: a systematic review protocol.* BMC research notes, 2010. **3**(1): p. 250.
35. Breitenstein, S.M. and D. Gross, *Web-Based Delivery of a Preventive Parent Training Intervention: A Feasibility Study.* Journal of Child and Adolescent Psychiatric Nursing, 2013. **26**(2): p. 149-157.
36. Love, S.M., et al., *Enhancing accessibility and engagement in evidence-based parenting programs to reduce maltreatment: Conversations with vulnerable parents.* Journal of public child welfare, 2013. **7**(1): p. 20-38.
37. Bigelow, K.M., J.J. Carta, and J. Burke Lefever, *Txt u ltr: Using cellular phone technology to enhance a parenting intervention for families at risk for neglect.* Child maltreatment, 2008. **13**(4): p. 362-367.
38. Gazmararian, J.A., et al., *Text4baby program: an opportunity to reach underserved pregnant and postpartum women?* Maternal and child health journal, 2014. **18**(1): p. 223-232.
39. Kharbanda, E.O., et al., *Text4Health: a qualitative evaluation of parental readiness for text message immunization reminders.* American journal of public health, 2009. **99**(12): p. 2176-2178.
40. Bramley, D., et al., *Smoking cessation using mobile phone text messaging is as effective in Maori as non-Maori.* 2005.


**©National Institute for Health Innovation, The University of Auckland, 2018** 

Confidential
Not for Public Circulation or Reproduction

41. Rodgers, A., et al., *Do u smoke after txt? Results of a randomised trial of smoking cessation using mobile phone text messaging.* Tobacco control, 2005. **14**(4): p. 255-261.
42. Zealand, R.N., *New Zealanders' Use of Mobile Electronic Devices*. 2014, Research New Zealand: Wellington, New Zealand.
43. Murphy, R. and M. Shepherd, *Diabetes Text-Message Self-Management Support Program (SMS4BG): A Pilot Study.*


**©National Institute for Health Innovation, The University of Auckland, 2018**

Confidential
Not for Public Circulation or Reproduction

# 14. Appendix 1 – Terms of Reference

## 14.1 Steering Group Committee

The Steering Group Committee will consist of the National Science Challenge: A Better Start Cure Kids grant application investigators and will be responsible for providing strategic guidance for the trial including developing and maintaining the study design, approval of the protocol, statistical analysis, presentation and publication of results. The Committee will meet as required during study development from start-up to review problems and issues raised by the Study Management Committee.

## 14.2 Study Management Committee

The Study Management Committee will be responsible for the daily operation of the study, and will develop study materials, deal with study problems, recruitment, and logistical issues. Meetings will be held weekly while the study is in development, then as required when the study is underway.


©National Institute for Health Innovation, The University of Auckland, 2018

# 15. Appendix 3 – Proposed Timeline

## 15.1 Trial Timeline

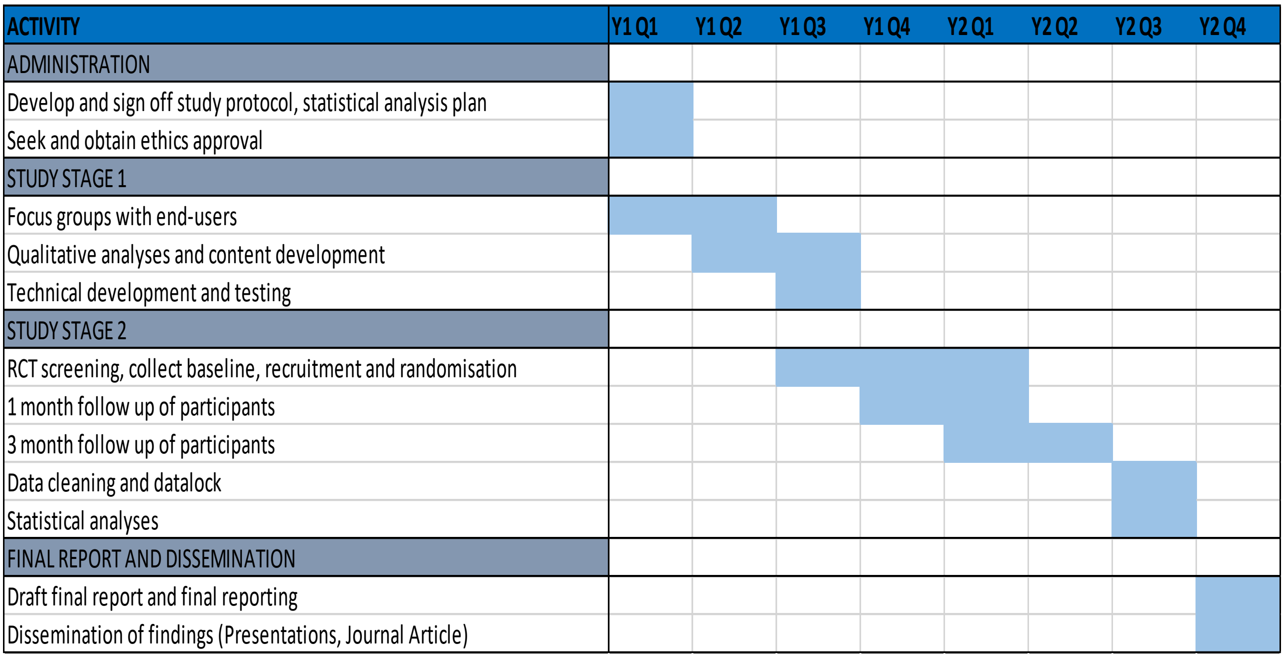


## 15.2 Key milestones

| **Date** | **Milestone** |
|---|---|
| Jul 2017 | Grant announced |
| Sept 2017 | Submit and obtain ethics approval |
| Sept 2017 | Contract between Cure Kids and University finalised |
| Oct 2017 | Research Assistant appointed |
| Oct – Nov 2017 | Focus group with end users |
| January 2018 | Technical development and testing completed |
| Feb 2018 | Recruitment begin |
| Sept 2018 | Recruitment completed (n =214, 30% Māori, 30% Pacific) |
| Dec 2018 | 3-Months Follow up Completed |
| Jan 2019 | Data lock |
| March 2019 | Data analysis complete |
| June 2019 | Results submitted for publication |
| June 2019 | Final report completed |


**©National Institute for Health Innovation, The University of Auckland, 2018**

Confidential
Not for Public Circulation or Reproduction

# 16. Appendix 4 – Summary of Protocol Amendments

| Date | Page | Section heading | Amendment |
|---|---|---|---|
| 14/12/18 | 9 | Exclusion criteria: | From: …. the Parental Stress Index (i.e., > 45) at screening will be excluded and directed to professional services.<br>To: …… the Parental Stress Index (i.e., ≥ 72) at screening will be excluded and directed to professional services. |
| 14/12/18 | 17 | 16.1.1 Exclusion criteria | From: Parents that show high level of stress as reported by the Parental Stress Index (i.e., ≥ 45) at screening will be excluded and directed to professional services.<br>To: From: Parents that show high level of stress as reported by the Parental Stress Index (i.e., ≥ 72) at screening will be excluded and directed to professional services. |
| | | | |
| | | | |
| | | | |
| | | | |
| | | | |
| | | | |
| | | | |
| | | | |
| | | | |
| | | | |
| | | | |
| | | | |
| | | | |
| | | | |
| | | | |
| | | | |


©National Institute for Health Innovation, The University of Auckland, 2018
