# Supplementary material for: Effect of MyTeen SMS-Based Mobile Intervention for Parents of Adolescents: A Randomized Clinical Trial
Source: JAMA Netw Open. 2019 Sep 11;2(9):e1911120. doi: 10.1001/jamanetworkopen.2019.11120 (PMC6739724; doi:10.1001/jamanetworkopen.2019.11120)
Supplement: Supplement 2. — eTable. Intervention Content Including the Domains, Strategies and Sample Text-Messages [file jamanetwopen-2-e1911120-s002.pdf]

Supplementary Online Content

Chu JTW, Wadham A, Jiang Y, et al. Effect of MyTeen SMS-based mobile intervention for parents of adolescents: a randomized clinical trial. *JAMA Netw Open*. 2019;2(9):e1911120. doi:10.1001/jamanetworkopen.2019.11120

eTable. Intervention Content Including the Domains, Strategies and Sample Text-Messages

This supplementary material has been provided by the authors to give readers additional information about their work.

© 2019 Chu JTW et al. *JAMA Network Open.*

eTable. Intervention content including the domains, strategies and sample text-messages.

Number of text messages per domain	Domains	Parenting Strategies	Sample Text Message
5	Establish and maintain a good relationship with your teenager	Show affection	Show your teen love and affection, talk with them and have fun with them! By doing so you're making a huge contribution to their emotional wellbeing.
		Take time to talk	Be sure to check in everyday. Few minutes each day while you're cleaning up after dinner/right before bedtime can keep you tuned in & establish open communication.
		Talk about emotions	Its common for teens to experience strong emotions. Keep calm & acknowledge the emotion. Help them to understand & express how they are feeling.
4	Be involved and support increasing autonomy	Be involved in your teenager's life	Spend quality time together. Don't get caught up in to-do lists & screens. Have a meal, take a walk, play a game, or make a list of things to do together.
		Support increasing autonomy	Include your teen! Let them make decisions, and ask for their opinion. They may say no most of the time, but don't stop asking.
3	Minimise conflict in the home	Minimising conflict and being a role model	Minimise conflict in the home and remember you are the role model for your teen! Stay calm & encourage them to find solutions to problems/conflict.
2	Encourage good health habits	Setting up healthy sleep habits	Sleep is really important for your teen! Encourage them to switch off/reduce the time they spend on their phone or devices a few hours before bedtime.
		Encouraging healthy diet and exercise	Eating well, regular exercise can improve your teen's mood, energy levels & general wellbeing. Encourage & teach them to set up healthy habits for their future.
5	Knowledge and risk of depression	Understanding depression	Depression = feelings of sadness & irritability lasting longer than 2 weeks, affect daily life and stop

© 2019 Chu JTW et al. *JAMA Network Open.*

			people from taking part in things they used to enjoy.
		Recognising symptoms	Don't ignore or assume it's typical teen moodiness. Ask questions, "I've noticed you've been really quiet & not hanging out with friends lately. What's going on?"
3	Encourage professional help seeking when needed	Know what help is available	If u need some help or support, don't hesitate to ask. Local GP, Parent Helpline: 0800 568 856, Family Services: 0800 211 211, http://www.commonground.org.nz/
		Seek help for depression	Help your teen find options. Encourage them to talk to friends/whanau & let them know about other support services. Call/Txt 1737 or Youthline: 0800 376 633
4	Parental self-care	Looking after yourself as a parent	Take care of yourself!!! Take a break, and get support for yourself too. If your own needs are met, its easier to be patient, consistent & available to your teen.
		Social support	Share the load – you're not expected to do miracles. Take a team approach - whānau, friends, relatives & support professionals. U don't have to do it all alone. (Emoji)

Note. Total of 28 messages were delivered, including a welcoming and closing message.

© 2019 Chu JTW et al. *JAMA Network Open.*
