# Supplementary material for: Effect of MyTeen SMS-Based Mobile Intervention for Parents of Adolescents: A Randomized Clinical Trial
Source: JAMA Netw Open. 2019 Sep 11;2(9):e1911120. doi: 10.1001/jamanetworkopen.2019.11120 (PMC6739724; doi:10.1001/jamanetworkopen.2019.11120)
Supplement: Supplement 3. — Data Sharing Statement [file jamanetwopen-2-e1911120-s003.pdf]

Data Sharing Statement

Chu JTW, Wadham A, Jiang Y, et al. Effect of MyTeen SMS-based mobile intervention for parents of adolescents: a randomized clinical trial. *JAMA Netw Open*. 2019;2(9):e1911120. doi:10.1001/jamanetworkopen.2019.11120

Data
Data available: Yes
Data types: Deidentified participant data
How to access data: All requests for de-identified individual participant data or study documents will be considered, after publication of the results, where the proposed use aligns with public good purposes, does not conflict with other requests, or planned use by the Study Steering Committee, and the requestor is willing to sign a data access agreement. Contact will be via the corresponding author (jt.chu@auckland.ac.nz).
When available: With publication

Supporting Documents
Document types: None

Additional Information
Who can access the data: All requests for de-identified individual participant data or study documents will be considered.
Types of analyses: Data will be made available if the proposed use aligns with public good purposes, does not conflict with other requests, or planned use by the Study Steering Committee.
Mechanisms of data availability: Data will be made available with a signed data access agreement.
